# MicroRNA-mediated regulation of the immune response in Calu-3 cells infected with a SARS-CoV-2 E gene variant

**DOI:** 10.3389/fmicb.2025.1643588

**Published:** 2025-12-15

**Authors:** Chenfan Sun, Fang Xu, Zhongji Pu, Han-Ping Zhu, Yu-dong Li, Hang-Jing Lu, Bei-Bei Wu, Yi-Sheng Sun, Ping-Ping Yao, Jian-Ming Jiang

**Affiliations:** 1 Zhejiang Key Lab of Vaccine, Infectious Disease Prevention and Control, Zhejiang Provincial Center for Disease Control and Prevention, Hangzhou, China; 2 Xianghu Laboratory, Hangzhou, China; 3 Department of Biological Engineering, School of Food Science and Biotechnology, Zhejiang Gongshang University, Hangzhou, China

**Keywords:** SARS-CoV-2, E gene mutant, microRNA, immune response, virus infection

## Abstract

**Introduction:**

SARS-CoV-2, the pathogen of COVID-19, disrupts the alveolar epithelial barrier and triggers exacerbation of airway inflammation. The envelope (E) protein plays a key role in promoting epithelial damage and sustaining inflammation. We previously identified a SARS-CoV-2 variant (F8) containing a 12-bp deletion in the E gene. Compared to the 8X strain, which possesses the wild-type E gene, F8 could induce a higher expression of inflammatory factors in Calu-3 cells.

**Methods:**

This study analyzed the miRNA expression profiles in Calu-3 cells infected with either the F8 variant or the 8X strain. Quantitative Reverse Transcription Polymerase Chain Reaction (RT-qPCR) was employed to validate the differential expression of candidate miRNAs. Subsequent functional verification assays like ELISA were performed to elucidate the regulatory roles of these miRNAs in host signaling pathways, such as inflammatory responses.

**Results:**

We discovered that F8 infection significantly upregulated miR-361-3p and downregulated let-7b-5p. Furthermore, miR-361-3p regulated the PI3K-Akt pathway by directly targeting and inhibiting TSPAN1, ultimately promoting PTEN expression. The downregulation of let-7b-5p might release the suppression on inflammatory cytokines (IL-6, IL-8, PTX3), and partially disorder ZO-1 expression in maintaining the barrier function.

**Discussion:**

This study is the first to demonstrate that the SARS-CoV-2 E protein mutant F8 remodeled the host miRNAs network to coordinate the immune responses and barrier function. All findings provided valuable insights into the pathogenesis of SARS-CoV-2 variants.

## 1 Introduction

Over the past two decades, three highly pathogenic coronaviruses (CoVs) have emerged to pose significant threats to humans, including the 2002 Severe Acute Respiratory Syndrome Coronavirus (SARS-CoV), the 2012 Middle East Respiratory Syndrome Coronavirus (MERS-CoV), and SARS-CoV-2, the etiological agent of the ongoing COVID-19 epidemic (Kesheh et al., 2022; Piccioni et al., 2023). These viruses, belonging to the β-coronavirus genera, cause severe respiratory tract infections and can lead to acute respiratory distress syndrome. As enveloped viruses, CoVs possess a single-stranded positive-sense RNA genome encoding four structural proteins: nucleocapsid (N), membrane (M), spike (S), and envelope (E) proteins (Zhu et al., 2020). The E protein is a small transmembrane protein composed of 75–109 amino acids, with a molecular weight of 8–12 kDa (Cao et al., 2021). Although present at low level in viral particle, the E protein highly expresses in infected cells and contains three structural domains: a N-terminal hydrophilic domain (NTD), a transmembrane domain (TMD), and a C-terminal domain (CTD). It maintains viral particle structure through interactions with the M protein and oligomerization to form cation-selective ion channels. Most E proteins are located at the Golgi apparatus, endoplasmic reticulum (ER), and ER-Golgi intermediate compartment (ERGIC) of infected cells (Zhou et al., 2023).

Notably, the E protein is a multifunctional structural protein that plays critical roles in viral assembly, budding, and membrane curvature during infection (Wong and Saier, 2021). As a transmembrane protein with ion channel activity, the E protein interacts with host cellular machinery to facilitate viral particle formation and release. Recent studies have highlighted its involvement in ERGIC trafficking, where it modulated lipid raft organization and promoted virus-like particle production (Boson et al., 2021). Structural analysis has revealed that the amphipathic α-helix domain is essential for membrane association and oligomerization, which is crucial for efficient viral budding (Kuzmin et al., 2022). Despite its conserved role in viral replication, the E protein's functional diversity across CoVs suggested its context-dependent contributions to pathogenesis.

The immunomodulatory effects of the E protein remain highly controversial. The E protein triggers host inflammatory responses by activating the NLRP3 inflammasome, leading to the secretion of IL-1β, TNF-α, and IL-6, cytokines critical for pathogen sensing and pathogenicity (Eisfeld et al., 2021; Yalcinkaya et al., 2021). Other reports also demonstrated that the E protein could induce inflammatory responses via interaction with TLR2 directly, promoting the production of pro-inflammatory cytokines, and inhibition of TLR2 conferring protection against SARS-CoV-2-mediated lethality (Zheng et al., 2021). Meanwhile, immune cells without ACE2 receptors could be infected by SARS-CoV-2 through TLR1 receptors. TLR1 receptors not only recognized E and M proteins but could also directly activate inflammatory signaling pathways (Duan et al., 2024). During respiratory virus infections, dysregulation in tight junction protein expression may impair the barrier function, allowing invading pathogens to enter the sub-epithelial space (Linfield et al., 2021). Some studies in mouse models have indicated that the E protein would induce tissue damage during infection (Zheng et al., 2021; Xia et al., 2021). Meanwhile, the E protein could suppress the expression of tight junction proteins, leading to disruption of the airway epithelial barrier (Xu et al., 2024). Interestingly, SARS-CoV with an E protein lacking ion channel activity exhibited significantly reduced lung epithelial damage, inflammation, and mortality in mice, despite similar levels of viral replication (Nieto-Torres et al., 2014; Shepley-McTaggart et al., 2021), which shared 96% identity with SARS-CoV-2. This dual functionality may explain why E protein mutations in circulating variants exhibited divergent effects on host immune responses. These discrepancies underscore the need to investigate how E protein sequence variations influence its interaction with host regulatory networks.

MicroRNAs (miRNAs) are critical post-transcriptional regulators that fine-tune interactions by modulating both viral replication and antiviral immune responses. During viral infection, host miRNAs would directly target viral RNA genomes or regulate antiviral signaling pathways, whereas viruses evolve strategies to hijack or disturb host miRNAs network to favor their survival (Pfeffer et al., 2005; Bartel, 2018). For instance, hepatitis C virus (HCV) exploited miR-122 to stabilize its RNA genome (Jopling et al., 2005), while influenza A virus (IAV) upregulated miR-1290 to inhibit vimentin expression and retain vRNP in the nucleus, thereby enhancing viral replication (Huang et al., 2019). SARS-CoV-2 infection has been shown to alter host miRNA profiles, correlating with cytokine storm severity and metabolic reprogramming (Battaglia et al., 2021; Hill and Lukiw, 2022; Fossat et al., 2023). However, the viral E protein, a critical virulence factor, has been implicated in modulating host cell apoptosis and inflammation (Baral et al., 2023; Shteinfer-Kuzmine et al., 2024), yet its role in reshaping miRNAs-mediated regulatory networks remains unexplored.

Our previous studies identified a novel E protein variant F8 with a 4-amino acid deletion at the TMD (Sun et al., 2020). Despite comparable viral titer and infectivity to the wild-type strain 8X, F8 vaccine showed a higher immunogenicity in mice. Transcriptomic analysis further revealed upregulation of inflammatory cytokines (e.g., IL-6, IL-8) in F8-infected Calu-3 cells (Sun et al., 2022). miRNAs play a pivotal role in the regulation of viral infection and immune responses, and a thorough investigation into how miRNAs respond to viral infections and regulation pathways of immunity are important to uncover the pathogenic mechanisms of the SARS-CoV-2. Current researches primarily focused on the regulatory effects between host miRNAs in the context of SARS-CoV-2 infection. However, the regulatory impact of the E protein, a key pathogenic factor, on the expression and physiological functions of miRNAs remains largely unexplored. In this work, we found that F8 infection altered host miRNA profiles, particularly enhancing miR-361-3p and suppressing let-7b-5p. These miRNAs were predicted to target key immune regulators. While viral non-coding RNAs have been extensively studied, the role of host miRNAs in the immunomodulatory effects mediated by E protein remains poorly understood. This study aims to elucidate how the E protein variant reshapes miRNAs-mediated regulation, thereby providing insights into immunogenicity and pathogenic potential.

In this study, we investigated the mutant strain F8 with a 12-bp deletion in the E protein, resulting in a 4-amino acid deletion in the E protein sequence. Our analysis revealed that F8 infection induced a distinct miRNAs expression profile in human lung adenocarcinoma Calu-3 cells, characterized by significant upregulation of miR-361-3p and downregulation of let-7b-5p. Mechanistic studies demonstrated that miR-361-3p activated the tumor suppressor gene PTEN through inhibition of transmembrane protein 1 (TSPAN1), thereby enhancing host type I interferon responses. Simultaneously, the downregulation of let-7b-5p relieved transcriptional suppression on inflammatory cytokines including IL-6, IL-8, and PTX3, while also might upregulate the tight junction protein ZO-1 to facilitate the maintenance of epithelial barrier integrity. These findings highlighted the critical role of E protein-mediated miRNAs regulation in coordinating antiviral immunity and epithelial homeostasis during viral infection.

## 2 Materials and methods

### 2.1 Virus and cell culture

The SARS-CoV-2 clinical strains F8 and 8X were purified from a male COVID-19 patient in Hangzhou (Sun et al., 2022, 2020). Calu-3 cells were purchased from the National Collection of Authenticated Cell Cultures. Cells were cultured in DMEM (Gibco, United States) containing 10% fetal bovine serum (FBS, Gibco, United States) at 37 °C in an incubator with 5% CO_2_. We seeded Calu-3 into 6-well plates at a density of 1 × 10^6^ cells/well, where F8 and 8X infection were worked at a multiplicity of infection (MOI) of 2. After incubation at 37 °C for 1 h, cells were gently washed with PBS and maintained in the virus growth medium (DMEM containing 3% FBS) for 48 h incubation. Harvest cells and the total RNA and miRNA were extracted by using a RNeasy Plus Mini Kit and miRNeasy Kit for miRNA Purification, respectively (QIAGEN).

### 2.2 Prediction of protein homodimer structures

To investigate the dimerization stability of the two protein sequences, we employed Protenix, a trainable PyTorch reproduction of AlphaFold 3 (ByteDance AML AI4Science Team et al., 2025; Zhou et al., 2025). Protenix was selected for its comprehensive capabilities in predicting protein-protein interactions and multimeric assemblies.

E_8X: MYSFVSEETGTLIVNSVLLFLAFVVFLLVTLAILTALRLCAYCCNIVNVSLVKPSFYVYSRVKNLNSSRVPDLLV.

E_F8: MYSFVSEETGTLIVNSVLLFLAFVVTLAILTALRLCAYCCNIVNVSLVKPSFYVYSRVKNLNSSRVPDLLV.

The primary structural difference between these sequences is the absence of four residues (FLLV) in the second sequence. For structure prediction, we followed the protocol described in the Protenix repository's “inference_demo.sh” script. For each sequence, we generated homodimeric assemblies using the following parameters: (a) five independent model samples with different random seeds. (b) 200 diffusion model steps for each prediction. (c) 10 cycles of backbone inference iterations. The stability of the predicted homodimers was evaluated using multiple metrics provided by Protenix.

We used Molecular dynamics (MD) simulation to determine which homodimer exhibits greater stability. MD simulation was conducted using NAMD 3.0.2 software and CHARMM36m force field (Phillips et al., 2020). The simulation employed periodic boundary conditions and the particle mesh Ewald method. The specific simulation process included equilibration of water molecules for 1 ns under the NVT ensemble, gradual heating from 0K to 300K, followed by 50 ns of productive molecular dynamics simulation under the NPT ensemble at 300K. The simulation trajectories were analyzed using Bio3D (Grant et al., 2021).

### 2.3 miRNA sequencing

The experimental steps for cell miRNA sequencing strictly adhere to the standard protocols provided by Illumina. The process mainly encompasses library preparation and sequencing. Three biological replicates were utilized. For small RNA sequencing library preparation, the TruSeq Small RNA Sample Prep Kits (Illumina, San Diego, USA) are employed. This preparation involves two crucial adapter ligation steps: first, ligating the 3′ and 5′ RNA adapters to the RNA molecules. Subsequently, reverse transcription PCR is carried out on the small RNAs with 3′ and 5′ adapters attached, in order to create a DNA library. After that, the amplified cDNA library undergoes gel purification to obtain high-quality library fragments. Once the library preparation is completed, the constructed library is sequenced using the Illumina Hiseq2500. The sequencing is performed with a single-end read length of 1 × 50 bp, ensuring accurate and efficient detection of miRNA sequences. The sequence data has been deposited in NCBI under SRA submission PRJNA1273436.

### 2.4 miRNA-Seq data analysis

Raw sequencing reads were processed using the ACGT101-miR pipeline (LC Sciences, Houston, USA) to eliminate adapter dimers, low-quality sequences, non-coding RNAs (rRNA/tRNA/snRNA/snoRNA), and repetitive elements. After removing adapters and low-quality reads, an average of 6 million clean reads were retained for subsequent analysis. Sequences of 18–26 nucleotides were aligned to species-specific precursors in miRBase 22.0 via BLAST, permitting 3′/5′ end variations and ≤ 1 internal mismatch. Known miRNAs were identified as sequences matching mature miRNAs in annotated hairpin arms, while novel miRNA candidates (3p/5p variants) were defined as sequences mapping to the complementary arm of established precursors. Unmapped sequences were cross-referenced with non-species-specific miRBase precursors and genomically localized through BLAST against the reference genome. Putative novel miRNAs were predicted through secondary structure analysis using RNAfold (http://rna.tbi.univie.ac.at), requiring: stem-loop structure (≥50 nt total, ≤ 20 nt loop), stable stem region (≥16 bp, ≤ 12 nt bulge), favorable thermodynamics (ΔG ≤ −15 kcal/mol), and mature miRNA constraints (≥12 bp in stem, ≥80% stem alignment, ≤ 8 nt/ ≤ 4 mismatches/ ≤ 2 bulges in mature region).

### 2.5 Target gene prediction

The significant differential miRNAs were predicted for their target genes using TargetScan (v5.0) and miRanda (v3.3a). The predicted target genes from both software were filtered according to the scoring criteria of each. In the TargetScan algorithm, target genes with a context score percentile less than 50 were excluded, while in the miRanda algorithm, target genes with a maximum free energy (Max Energy) greater than −10 were excluded (i.e., the threshold being TargetScan score ≥ 50, miRanda Energy < −10). Finally, the intersection of these two software programs was taken as the final target genes for differential miRNAs.

### 2.6 KEGG and GO enrichment analysis

The predicted target genes were analyzed for pathway enrichment using the Kyoto Encyclopedia of Genes and Genomes (KEGG) and Gene Ontology (GO) databases. Firstly, all selected miRNA corresponding target genes are matched to the number of genes annotated for each function or pathway. Then, hypergeometric testing is applied to identify significant enrichment of functions related to the target genes of all selected miRNA compared to the gene count associated with the GO or KEGG pathways in the annotation database. *p*-value ≤ 0.05 is calculated as the threshold for significance, with functions meeting this condition defined as significantly enriched in the miRNA-mRNA relationship.

### 2.7 RT-PCR

RNA was reverse transcribed into cDNA using a reverse transcription kit (Vazyme, Inc.), where miRNA reverse transcription employed specific primers. The expression levels of target genes were detected using One Step TB Green^®^ PrimeScript™ PLUS RT-PCR Kit (Takara Bio, Inc.). For normalization, GAPDH was used for target genes, while U6 served as the endogenous control for miRNAs detection. The primer sequences are presented in Supplementary Table S1. The PCR conditions were set as follows: 10 min at 95 °C, followed by 40 cycles of 5 s at 95 °C and 34 s at 60 °C. Data were calculated using the 2^−Δ*ΔCq*^ method.

### 2.8 zsGreen1 reporter assay

The potential targets of miR-361-3p and the main binding sites between miR-361-3p and TSPAN1 were predicted using TargetScan (http://www.targetscan.org/). The 3′UTR sequences containing the wild-type (WT) or mutant (mut) binding sites of miR-361-3p and TSPAN1 were inserted into the pcDNA3.1-pCMV-zsGreen1 vector, replacing the original bGH polyA tail, constructing the zsGreen1 green fluorescent reporter plasmids. The reporter plasmids were co-transfected with either the miR-361-3p expression plasmid or vector into Calu-3 cells using Lipofectamine 3000 (Vazyme, TL301). 48 h after transfection, the green fluorescence intensity was analyzed using a fluorescence microscope and a Tecan Spark multimode microplate reader.

### 2.9 Detection of IL-6, IL-8 in supernatant of Calu-e cells

Cytokines IL-6, IL-8 were measured in the supernatant of Calu-3 by ELISA. We used the human IL-6 ELISA Kit (High-sensitive; PI325, Beyotime) and human IL-8 ELISA Kit (High-sensitive; PI641, Beyotime) to quantify. The experimental procedures were carried out according to the instructions provided in the manual.

### 2.10 Statistical analysis

All the statistical analysis in this work were performed in GraphPad Prism 9. The *t* test was used to compare the samples in two groups, and ANOVA was used for samples in three groups. Significance meant *p* value < 0.05.

## 3 Results

### 3.1 Enhanced homodimer stability in E protein mutant of F8 strain

**Figure 1:**
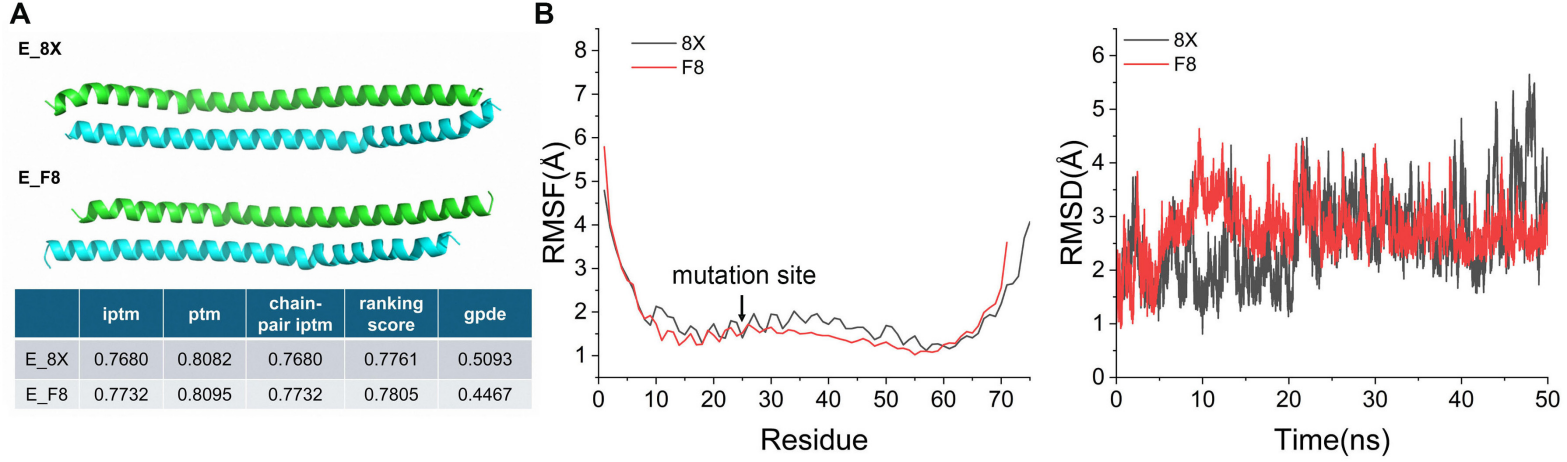
Prediction of homodimer structures of the E protein in F8 and 8X strains. **(A)** Interface Predicted TM-score (iptm): Measures the confidence of interface prediction between chains. Predicted TM-score (ptm): Evaluates the overall quality of the folded structure. Chain Pair Interface Predicted TM-score (chain-pair iptm): Specifically assesses the strength of the inter-chain interactions. Global Predicted Distance Error (gpde): Estimates the global structural error in the prediction. Higher values of iptm, ptm, and chain-pair iptm indicate better quality, while lower gpde values suggest more accurate predictions. **(B)** MD results of homodimer of the E protein in F8 and 8X strains. RMSF: Root Mean Square Fluctuation. RMSD: Root Mean Square Deviation.

The E protein is the smallest structural protein in the virion. Five E proteins assemble to form a pentameric transmembrane ion channel, which induces an inflammatory response in infected cells. A structural model revealed that the mutant, lacking residues FLLV (E_F8), exhibited superior dimer stability compared to wild-type E_8X (Figure 1A). The predicted local distance difference test (pLDDT), representing the confidence of a single chain, was 83.0 for both proteins, and the ptm values of F8 and 8X were 0.8095 and 0.8082, respectively. This indicated that the prediction quality of the monomeric structure and the overall structure were similar.

Compared to 8X, E_F8 exhibited significant advantages in both dimerization interface and overall conformational stability. Key metrics including iptm and gpde indicated a well model confidence. The very small differences between WT and F8 (e.g., ptm 0.8095 vs. 0.8082) were within expected model variation. MD simulation was conducted to investigate the stability of the E protein homopolymer (Figure 1B). The RMSD values of E_F8 could reach and maintain stability more quickly, with its post-equilibrated average slightly lower than E_8X. Conversely, the mutant E_F8 exhibited lower RMSF values in the region following the mutation site compared to E_8X, where contained TMD and partial CTD close to it. Therefore, we speculated that, in comparison to the wild-type E_8X, the mutant E_F8 demonstrated higher structural stability in its homologous dimer. This stabilization effected likely originates from the F8 mutation restricting the conformational flexibility of its downstream region, thereby stabilizing the interaction interface of the dimer, and the F8 mutant might regulate the biological functions of the virus by this.

miRNAs are increasingly recognized as critical modulators of host immune responses during viral infection. To characterize the miRNA expression profiles induced by F8 infection, miRNA sequencing was performed in Calu-3 cells infected with F8, 8X, and a control without infection (F8, 8X, and ctrl). The miRNA sequencing and bioinformatic analysis pipeline were illustrated in Supplementary Figure S1A. To validate the biological relevance of the sequencing data, we analyzed the length distribution of the filtered reads. Most sequences fell within the range of 20–24 nt (Supplementary Figure S1B), a signature of Dicer enzyme cleavage during canonical miRNA biogenesis, confirming the accuracy and reliability of the miRNA identification process.

### 3.2 F8 infection induced distinct alterations in the miRNAs expression

**Figure 2:**
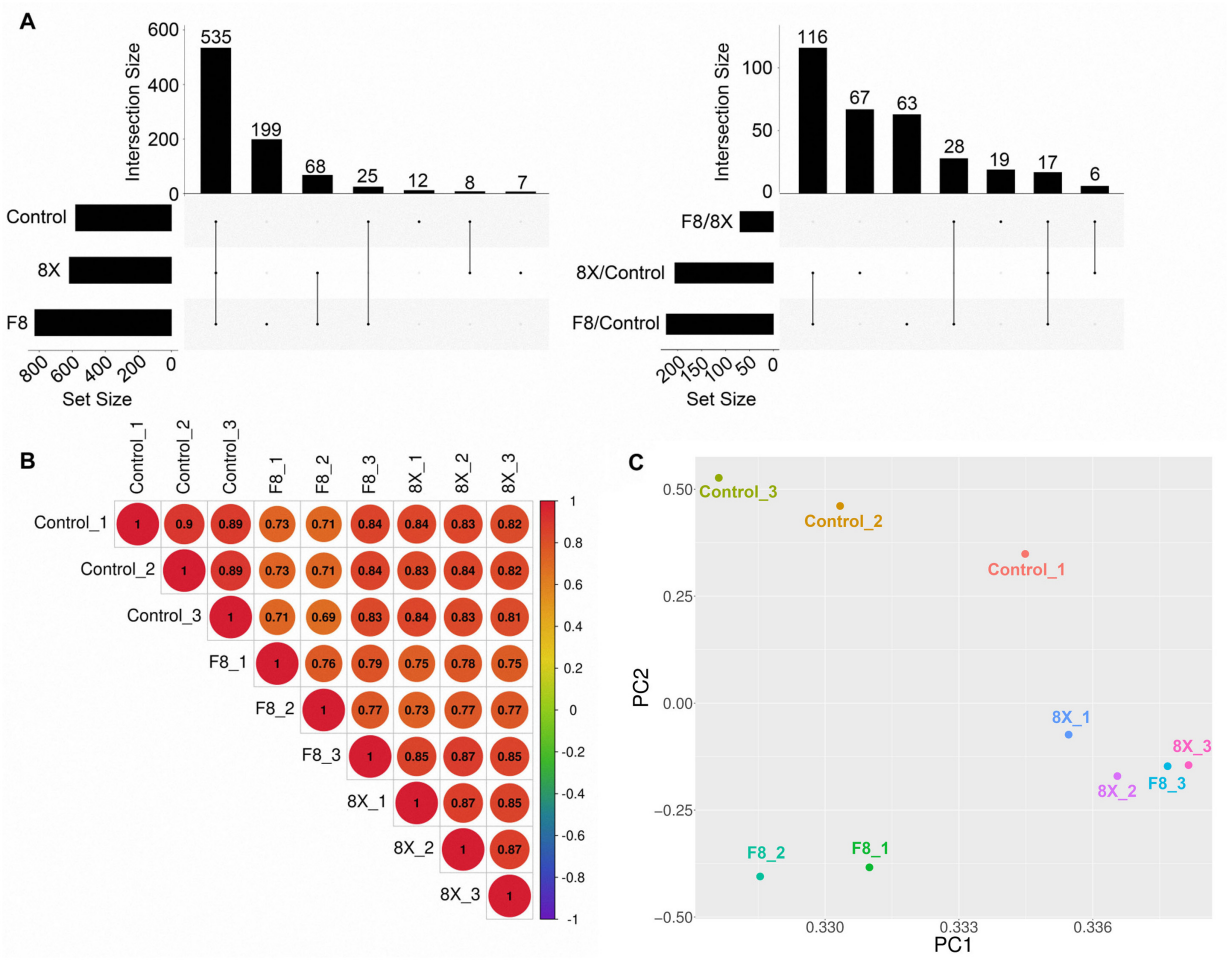
The basic characters of miRNAs identified in virus-infected Calu-3 cells. **(A)** UpSet plots showing the overlap between normal and viral-infected cells, and the overlap of differentially expressed miRNAs between 8X and F8, 8X and control, F8 and control. **(B,C)** Pearson correlation coefficient **(B)** and PCA plot **(C)** of miRNAs detection in each sample.

A total of 854 human miRNAs were identified in this study. Under our detection criteria, 199 miRNAs were specifically detected in the F8-infected group (Figure 2A, Supplementary Table S2). Both Pearson correlation coefficient analysis and principal component analysis (PCA) revealed a pronounced divergence of the F8 group from both 8X and control groups, with the lowest similarity to control group in Pearson correlation coefficients (Figure 2B). Only 17 different miRNAs were shared among three comparison groups (Figure 2A), and PCA further corroborated this trend, with F8-infected samples clustering distinctly from 8X along the first principal component (PC1: 96.16% variance explained) while only 2.9% variance was explained in PC2, distinguishing F8, 8X and control (Figure 2C). These results indicated that it was a unique miRNAs regulatory network resulting from E protein mutation, rather than the amplification of wild-type effects, that made the divergence between F8 and 8X groups.

### 3.3 Bioinformatic prediction revealed that F8 infection might affect immune signaling via differential miRNAs regulation

**Figure 3:**
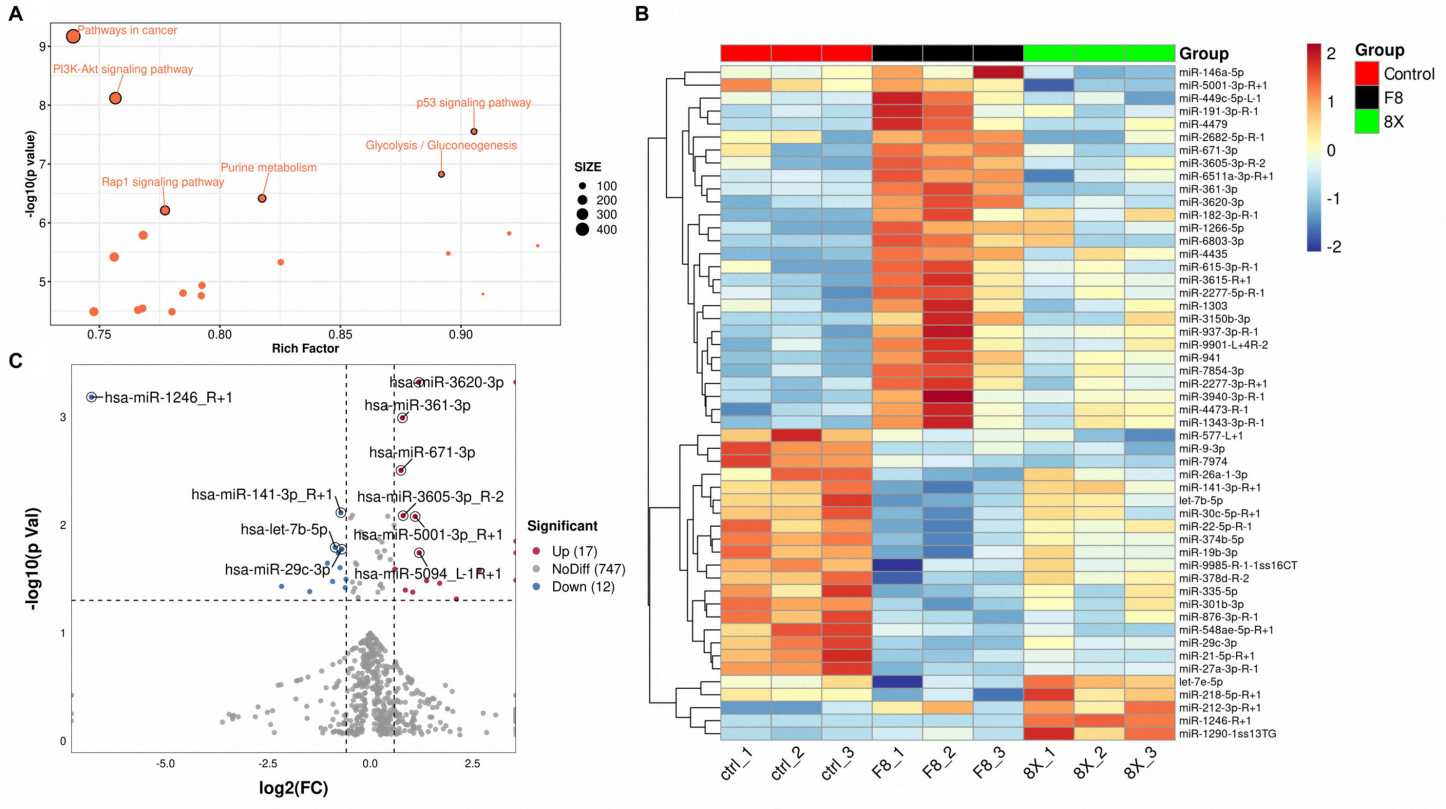
Differentially expressed miRNAs in virus-infected and contrasted Calu-3 cells. **(A)** KEGG enrichment analysis of host DE miRNA targets. **(B)** Heat maps for differentially expressed miRNAs in three groups. **(C)** Volcano plots for differentially expressed miRNAs between F8 and 8X infected Calu-3 cells.

The 854 human-type target genes were subjected to GO functional analysis and differentially expressed miRNAs were analyzed through GO and KEGG enrichment. Most GO terms appeared unrelated to virus infection (Supplementary Figures S2, S3). In the KEGG enrichment analysis, we screened and presented the top 20 significantly enriched KEGG pathways (Figure 3A). Among these, the PI3K-Akt signaling pathway and the p53 signaling pathway were two of the most significant terms. Notably, the PI3K-Akt signaling pathway was also enriched in KEGG pathway analysis specifically on the genes predicted to be targeted by the 29 significantly altered miRNAs from the F8 vs. 8X comparison (Supplementary Figure S4), and it plays a crucial regulatory role in the cellular defense against viral infections. Based on this, we hypothesized that the E protein might influence the immune response process of Calu-3 cells by modulating the PI3K-Akt signaling pathway.

To elucidate the host miRNAs regulatory landscape induced by F8 infection, we conducted differential expression analysis with the criteria of *p* < 0.05 and fold change (FC) ≥ 1.5 or ≤ 0.67 (F8/8X ratio). Heatmap clustering analysis revealed a distinct segregation of the F8-infected group from both 8X-infected and control group (Figure 3B). This indicated that the E protein mutation drove a unique miRNA expression signature, with 17 miRNAs being upregulated and 12 downregulated. Compared with the 8X group, miR-361-3p (FC = 1.73, *p* = 0.00005) and let-7b-5p (FC = 0.55, *p* = 0.005) exhibited the significant differential expression (Figure 3C). Notably, miR-361-3p and let-7b-5p are functionally linked to the PI3K-Akt signaling pathway and inflammatory cytokine signaling, intersecting directly with the enriched pathways described above. To explore their roles in F8 infection, we prioritized these miRNAs for downstream validation, utilizing gain-of-function and loss-of-function experiments to investigate their regulatory impacts on host immune responses.

### 3.4 miR-361-3p inhibited TSPAN1 might influence the PTEN/PI3K-Akt pathway

**Figure 4:**
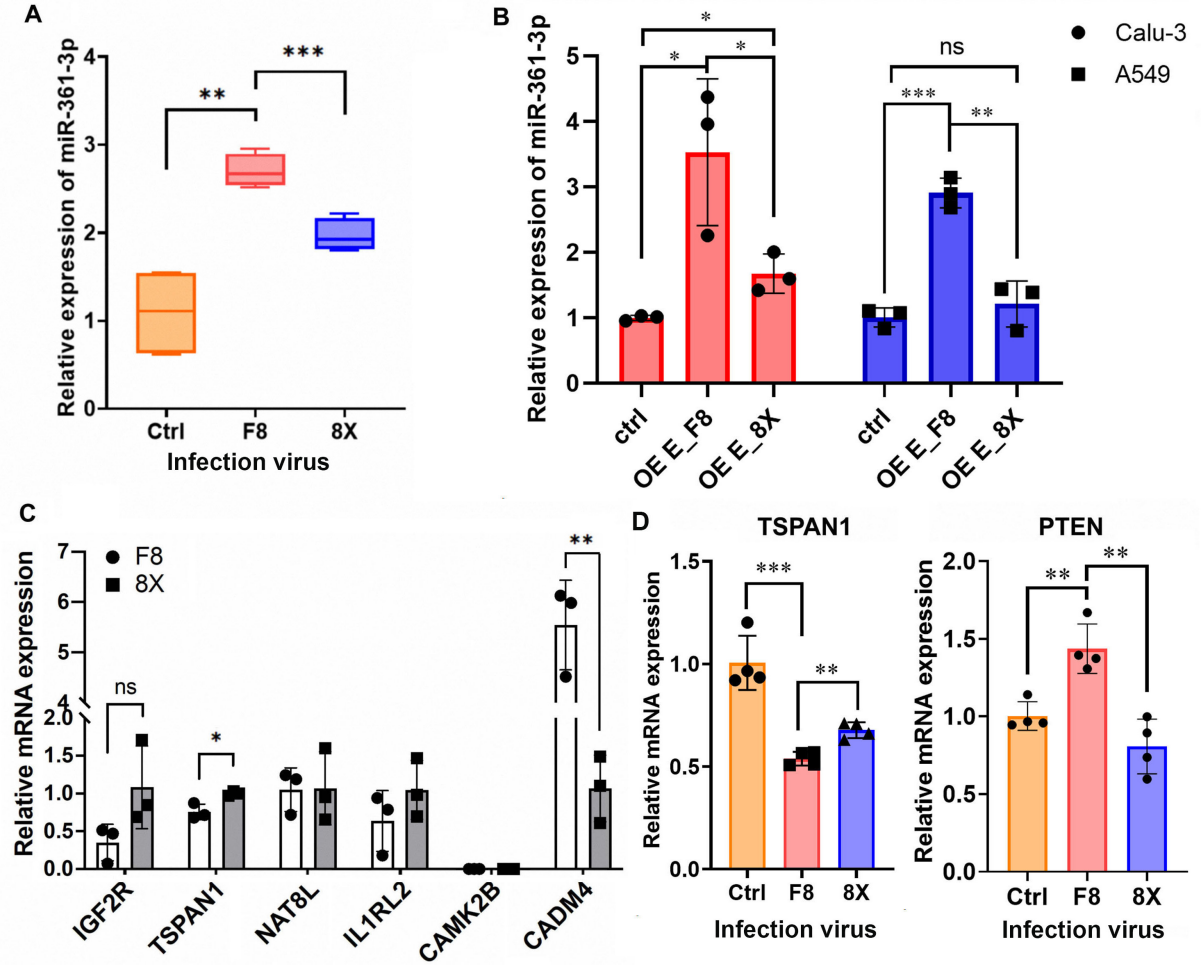
miR-361-3p was up-regulated during F8 infection and inhibited the transcription of TSPAN1. **(A)** miR-361-3p expression was detected by RT-qPCR in viral-infected (F8 and 8X) and normal Calu-3 cells (Ctrl). **(B)** miR-361-3p expression was detected by RT-qPCR in Calu-3 and A549 cells with overexpression of E_F8 and E_8X. **(C)** Transcription of putative targets of miR-361-3p in F8 and 8X infected cells. **(D)** qRT-PCR results of TSPAN1 and PTEN. *P < 0.05, **P < 0.01, ***P < 0.001, ns means no significant differences, as determined by unpaired *t*-test.

To elucidate the regulatory mechanism of miR-361-3p in F8 infection, RT-qPCR validation revealed that miR-361-3p expression was significantly upregulated in F8-infected Calu-3 cells compared with uninfected and 8X-infected cells (Figure 4A), same as the result of overexpression with two types of E protein in Calu-3 and A549 (Figure 4B). Using TargetScan for target gene prediction, six candidate target genes, including IGF2R (Zhang et al., 2021), TSPAN1 (Li et al., 2021), NAT8L, IL1RL2, CAMK2B and CADM4 were identified, and their mRNA levels were detected in F8 and 8X groups. The results showed that the transcription of TSPAN1 was significantly downregulated in the F8-infected group (Figures 4C, D), consistent with the negative regulatory effect of miR-361-3p on target genes.

Previous studies have confirmed that TSPAN1 activated the PI3K-Akt signaling pathway, leading to the inhibition of PTEN transcription (Wang et al., 2018). In line with this, the transcriptional level of PTEN was significantly increased in the F8-infected group (Figure 4D), suggesting that miR-361-3p would target and suppress on TSPAN1, thereby relieving its inhibitory effect on PTEN through regulation of the PI3K-Akt signaling pathway. This mechanism not only corroborated the enrichment of the PI3K-Akt pathway identified in the previous KEGG pathway analysis but also provided a potential molecular explanation for how the F8 mutant strain enhanced host antiviral immune responses, such as the interferon response.

**Figure 5:**
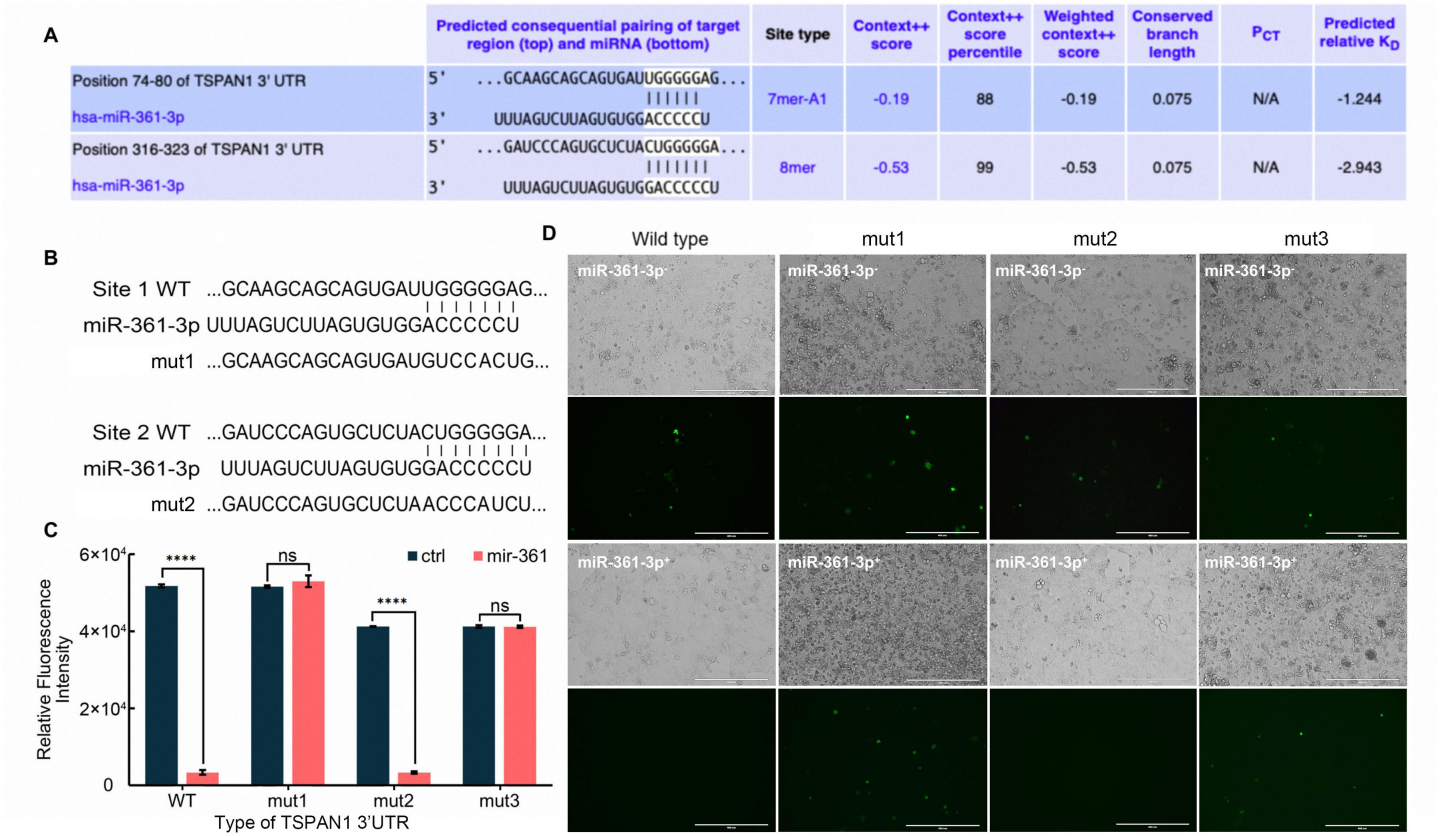
TSPAN1 acted as the direct target of miR-361-3p. **(A)** Putative binding sites of miR-361-3p on the 3′UTR region of TSPAN1 predicted by TargetScan. **(B)** Mutations were written bottom. **(C,D)** zsGreen1 detection by Tecan Spark multimode microplate reader **(C)** and fluorescence microscope **(D)**. Scale bar, 400 μm. The experiment was performed with *n* = 3 independent biological replicates, and that the data are presented as the mean ± SD. ****P < 0.0001, ns means no significant differences, as determined by unpaired *t*-test.

To characterize the regulatory mechanism of miR-361-3p targeting TSPAN1, we employed fluorescent reporter vectors containing zsGreen1 with wild-type (WT) and mutants of the *tspan*13′*UTR*. Based on two predicted miR-361-3p binding sites identified by TargetScan (Figure 5A), we constructed single mutation at site 1 (mut1), site 2 (mut2), and a double mutation with both mutations in site 1 and site 2 (mut3; Figure 5B). Co-transfection of miR-361-3p expression vector and reporter plasmids in Calu-3 cells showed that miR-361-3p could significantly suppress zsGreen1 fluorescence intensity driven by the wild-type *tspan*13′*UTR* plasmid. This suppressive effect was completely abrogated in the mut1 and mut3 (Figures 5C, D). In contrast, the result for the site 2 single-mutant (mut2) was consistent with the wild-type, with miR-361-3p expression significantly reducing fluorescence intensity (*p* < 0.0001). These results confirmed that site 1 was crucial for miR-361-3p binding and repression of TSPAN1 transcription in Calu-3 cells. We also testified this result in HEK293T cells (Gharib et al., 2024; Li et al., 2020), which were consistent with observations in Calu-3 cells (Supplementary Figure S5). Combined with our previous findings in F8-infected cells (Figures 4B, C), this study is the first to demonstrate that the E protein mutant might modulate the PTEN/PI3K-Akt pathway via the miR-361-3p/TSPAN1 axis, thereby participating in viral responses.

### 3.5 Let-7b-5p would suppress pro-inflammatory cytokine expression

**Figure 6:**
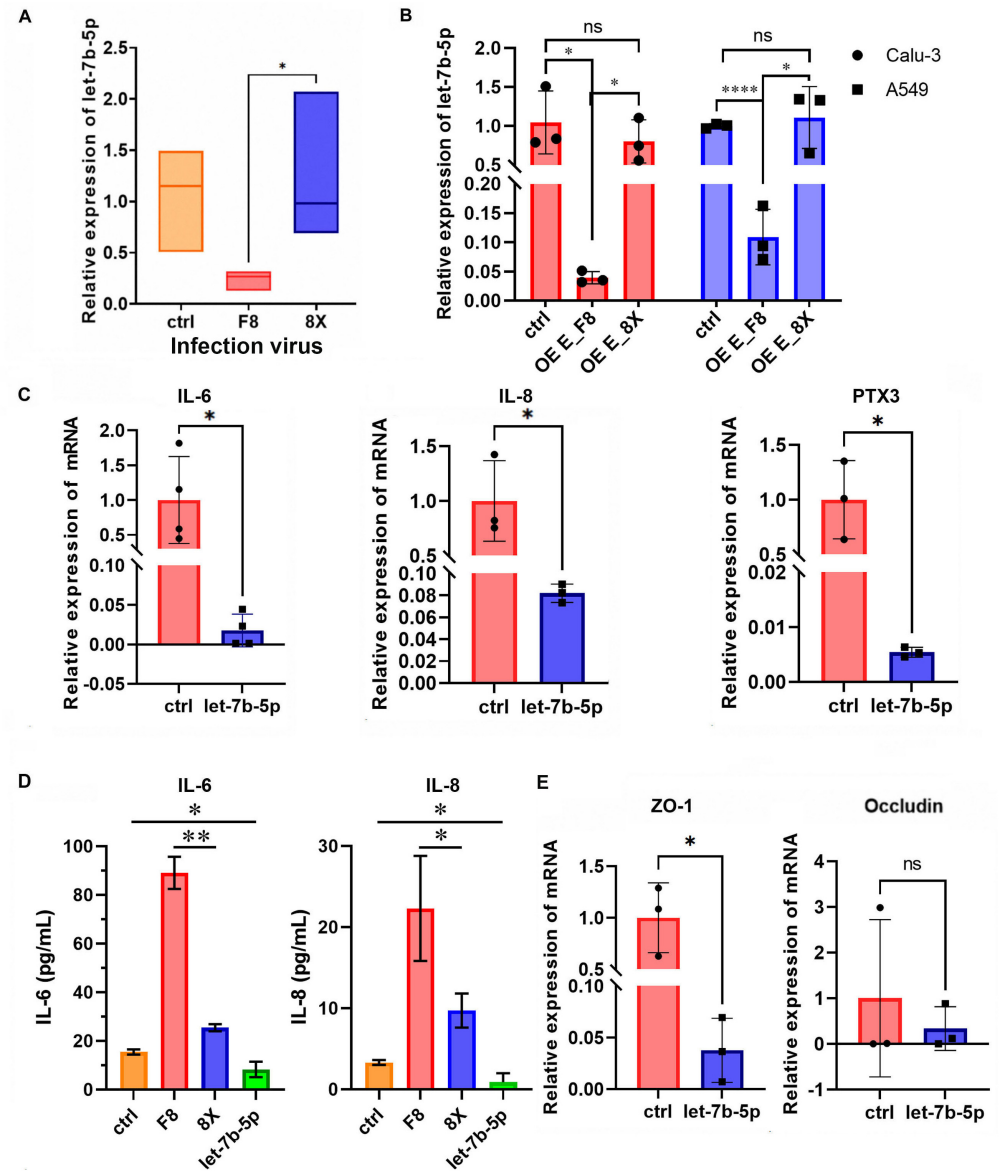
Let-7b-5p was inhibited in F8 infection and might release the suppression of pro-inflammatory cytokines. **(A)** Let-7b-5p expression was detected by RT-qPCR in Calu-3 cells with or without infection. **(B)** Let-7b-5p expression was detected by RT-qPCR in Calu-3 and A549 cells with overexpression of E_F8 and E_8X. **(C,E)** qRT-PCR results of IL-6, IL-8, PTX3 **(C)** and ZO-1, Occludin **(E)** in Calu-3 transfected with empty vector (ctrl) and let-7b-5p overexpression plasmid. **(D)** Concentration of IL-6 and IL-8 in the supernatant of Calu-3 with- or without infection and overexpression of let-7b-5p. *P <0.05, **P <0.01, ****P <0.0001, ns means no significant differences, as determined by unpaired *t*-test.

To systematically investigate the role of let-7b-5p in F8 infection, RT-qPCR analysis revealed that let-7b-5p expression was significantly downregulated in F8-infected Calu-3 cells compared with the 8X group (*p* < 0.05, Figure 6A). And overexpression of E variant in Calu-3 and A549 would also resulted in inhibitation of let-7b-5p, whereas no significant difference in E protein group (Figure 6B). Previous studies have shown that let-7b-5p could regulate host immune responses by targeting inflammatory signaling pathways such as TLR4/NF-κB signaling (Gong et al., 2022; Nematian et al., 2018). Meanwhile, in our previously study, it has been demonstrated that compared to the wild-type 8X strain, Calu-3 cells infected with F8 would express higher levels of IL-6, IL-8, and PTX3 (Sun et al., 2022). Consistent with these findings, overexpression of let-7b-5p significantly suppressed the expression of pro-inflammatory cytokines IL-6, IL-8, and PTX3 (Figures 6C, D), indicating that low let-7b-5p expression during F8 infection might cause pro-inflammatory effect by relieving inhibition of inflammatory cytokines indirectly. Previous research have indicated that E protein could lead to decreased expression of tight junction proteins, resulting in the disruption of the airway epithelial barrier (Xu et al., 2024). Meanwhile, there might be some relationship between let-7b-5p and epithelial barrier function (Gong et al., 2022). Consequently, we investigated whether F8 infection could also potentially impact epithelial barrier function through differential expression of miRNA. Notably, we identified a novel role for let-7b-5p in regulating the epithelial barrier protein ZO-1. Let-7b-5p overexpression suppressed ZO-1 transcription, whereas it had no significant effect on the expression of another tight junction protein, Occludin (Figure 6E). This selective regulation in Calu-3 cells indicated that the constitutive downregulation of let-7b-5p in F8 infection would contribute to the dysregulation of ZO-1, and might have an effect on epithelial barrier permeability.

## 4 Discussion

The E protein, a small transmembrane protein consisting of 75–109 amino acids with a molecular weight of 8–12 kDa, is modestly present in viral particles but highly expresses in infected cells (Cao et al., 2021; Zhou et al., 2023). It stabilizes the viral particle structure through interactions with the M protein and oligomerization to form cation-selective ion channels (Zhou et al., 2023). As a multifaceted structural component, the E protein is crucial for viral assembly, budding, and membrane curvature during infection (Wong and Saier, 2021). Its ion channel activity enables interaction with host cellular machinery to facilitate viral particle formation and release. Additionally, the E protein is involved in ERGIC trafficking, lipid raft configuration, and virus-like particle generation, with its amphipathic α-helix domain being essential for membrane association and oligomerization (Boson et al., 2021; Kuzmin et al., 2022). Although it is conserved in viral replication, the functional diversity of the E protein across CoVs suggests variable contributions to pathogenesis.

Although the role of the E protein in viral assembly has been widely studied, its impact in miRNAs remains unexplored. This study, for the first time, revealed that the SARS-CoV-2 variant F8 with a 12-bp deletion in E protein drove coordinated regulation in immune responses and barrier functions by remodeling the host miRNAs expression profile. E protein forms oligomers by monomer aggregation to construct ion channels. We predicted and analyzed the full-length structure and homodimer affinity of the wild type and E variant in F8 (Figure 1). The results indicated that The mutation of the E protein induces structural effects that restrict the atomic movements in its downstream regions, reducing conformational flexibility and enhancing the stability of local structures. This provided structural clues to explain the functional differences. However, the lack of high-resolution structure of full-length pentamers as precise templates posed a challenge to constructing reliable pentamer computational models.

Through miRNA sequencing and functional validation, we found that this E protein variant might influence PI3K-Akt signaling pathway, transcription of inflammatory cytokine and tight junction protein. This multidimensional regulatory paradigm highlighted a novel role of viral structural protein and the significance of miRNAs in viral-host interactions. PCA analysis indicated that the miRNAs transcriptome of Calu-3 cells were significantly different after F8- and 8X- infection. However, we observed that the miRNAs transcriptional response of one sample, F8_3, in the F8 group was closer to that of the 8X group (Figure 2C). In sample F8_3, there might be a compensatory pathway where some genes or pathways would response to balance the transcription of miRNAs compensating for the differential function of the E protein, resulting in a phenotype similar to the 8X group. This highlighted the robustness of biological systems. It deserves to be further developed and verified through additionally studies.

In this work, we demonstrated that F8 infection significantly upregulated miR-361-3p and inhibited let-7b-5p expression (Figure 3). By expressing the E protein and E variant individually in Calu-3 cells, we confirmed that the E variant would induce upregulation of miR-361-3p and downregulation of let-7b-5p. This finding replicates the trend of miRNA changes during F8 virus infection, establishing a causal relationship between E protein mutation and specific miRNA expression. miR-361-3p promoted PTEN transcription by inhibition on TSPAN1, mediated through the PI3K-Akt pathway in theory (Figure 4). Conversely, the downregulation of let-7b-5p favored inflammatory responses by relieving inhibition of IL-6, IL-8, and PTX3, while partially alleviating suppression of ZO-1 to maintain epithelial barrier function (Figure 6). These findings established the first connection between the coronavirus structural E protein and host miRNAs regulatory networks, providing a novel mechanism for understanding how viral virulence factors modulate host adaptability through miRNAs regulation.

Previous research have indicated that compared to mild coronaviruses (229E, HKU1, or OC43), The E protein of severe coronaviruses contains a distinctive SS/DS motif, which significantly enhances the release of inflammatory factors (Liu et al., 2024). These E proteins interacted with TMED10, activating the THU non-classical secretion pathway and exacerbating the release of inflammatory factors. In addition to exacerbating inflammation, the E protein could downregulate the expression of tight junction proteins ZO-1 and occludin, disrupting the airway epithelial barrier (Xu et al., 2024). In this experiment, we conducted a similar exploration focusing on a unique E mutant. Although the high expression of PTEN would theoretically limit the production of inflammatory cytokines, this work and preliminary results of this study (Sun et al., 2022) showed a significant upregulation of inflammatory cytokines such as IL-6 and IL-8 in the F8-infected group, which demonstrated the significant role of E protein in inflammatory responses once more (Figure 6D).

We speculated that the marked downregulation of let-7b-5p might be the dominant factor contributing to the enhanced inflammatory effect. Our findings confirmed that F8 infection leads to a significant decrease in the expression of let-7b-5p (Figure 6A), and the overexpression of let-7b-5p could effectively inhibit the transcription of IL-6, IL-8, PTX3 and ZO-1 (Figures 6C–E). The significant downregulation of let-7b-5p may coordinately drive two effects. First, it might indirectly relieve inhibition on IL-6, IL-8, and PTX3, promoting the release of inflammatory cytokines to enhance excessive inflammation and antibody responses. At the same time, alleviation of suppression on ZO-1 would partially preserve epithelial barrier function to balance the pathological damage caused by immune activation. Additionally, the inhibitory effect of PTEN on inflammatory cytokines might also be influenced by cell types. Therefore, the upregulation of inflammatory cytokines during F8 infection was a comprehensive result from pro-inflammatory effect dominated by the deficiency of let-7b-5p and the pleiotropy of PTEN function. This once again reflected the complex strategy employed by the virus to finely balance antiviral response and immune-pathological damage by regulating multiple miRNAs nodes in the host. Unfortunately, we haven't identified the effect of let-7b-5p and E protein variant on epithelial barrier function, which was only a hypothesis in this work.

In conclusion, it was the first time to demonstrate that the SARS-CoV-2 E protein mutant remodeled the host miRNAs networks to influence immune responses. The F8 infection significantly upregulated miR-361-3p and downregulated let-7b-5p, leading to a higher expression of inflammatory factors compared with the 8X-infected Calu-3 cells. These findings might lay foundation for better understanding the function of E protein and the pathogenesis of the SARS-CoV-2 variants. This novel mechanism by which specific virulence factors of the virus, like the E protein variant, had an effect on the host miRNA network to regulate the immunity and infection. This might provide potential new targets for future antiviral strategies. However, most results in this study were testified in transcription level, validation at the protein level should be done in the further study.

While this study provides evidence for the role of specific miRNAs in regulating host responses in Calu-3 cells, several limitations should be acknowledged. It is worth noting that many significant entries revealed by GO enrichment analysis do not have a direct association with typical viral infection pathways. This phenomenon might reflect the specific functional background of the host cell Calu-3 itself, with these fundamental cellular processes being widely disrupted when under viral attack. Meanwhile, Calu-3 cells originate from human lung adenocarcinoma. While it retains many characteristics of lung adenocarcinoma cells, the essence of its cancerous transformation determines significant differences from normal human lung epithelial cells in various aspects. These differences might influence the immunoregulation of the mutant E variant, which would be one of the directions for future research that we need to explore. At the same time, they may not fully recapitulate the complex physiology and architecture of primary human airway epithelium *in vivo*. Furthermore, the conservatism of this regulatory mechanism in the long-term effects of COVID-19 is also a subject that requires further investigation. We hope to validate in normal lung epithelial cells to exclude interference from the tumor background, and assess whether the observed alterations persist over the medium or long term. Not only can we answer the immune regulatory function of the E variant, but we can also delve into the role of tumor background in Calu-3, providing a more accurate and reliable assessment of the true biological function of the E variant in F8. Second, our findings were derived from an in vitro model with Calu-3 cells. Although this model is well-established for studying pathogenesis and barrier function of SARS-CoV-2 (Xu et al., 2024; Zhang et al., 2024), it cannot fully recapitulate the complexity of an *in vivo* environment, which includes immune cell interactions, systemic responses, and the multicellular architecture of respiratory tissue. Third, the 199 miRNAs, which was specifically detected in the F8-infected group, require further verification and functional analysis to elucidate their regulatory roles. Functional assays such as transepithelial electrical resistance or permeability assays, and animal models would be carried out to further substantiate our conclusion.

## Data Availability

The original contributions presented in the study are publicly available. This data can be found here: ncbi.nlm.nih.gov/bioproject/PRJNA1273436/.

## Supplementary material

Supplementary Table S1 Primers in this work.

Supplementary Table S2 All expressed miRNAs identified in this work.

Supplementary Figure S1 Workflow of miRNA sequencing.

Supplementary Figure S2 GO analysis of miRNAs.

Supplementary Figure S3 GO enrichment of different miRNAs between F8 and 8X.

Supplementary Figure S4 KEGG enrichment analysis on the genes predicted to be targeted by the significantly altered miRNAs from the F8 vs. 8X comparison.

Supplementary Figure S5 Identification for target site of miR-361-3p on 3′UTR of TSPAN1 in HEK293T.
