# Supplementary figures and images for: MicroRNA-mediated regulation of the immune response in Calu-3 cells infected with a SARS-CoV-2 E gene variant
Source: Front Microbiol. 2025 Dec 15;16:1643588. doi: 10.3389/fmicb.2025.1643588 (PMC12745296; doi:10.3389/fmicb.2025.1643588)

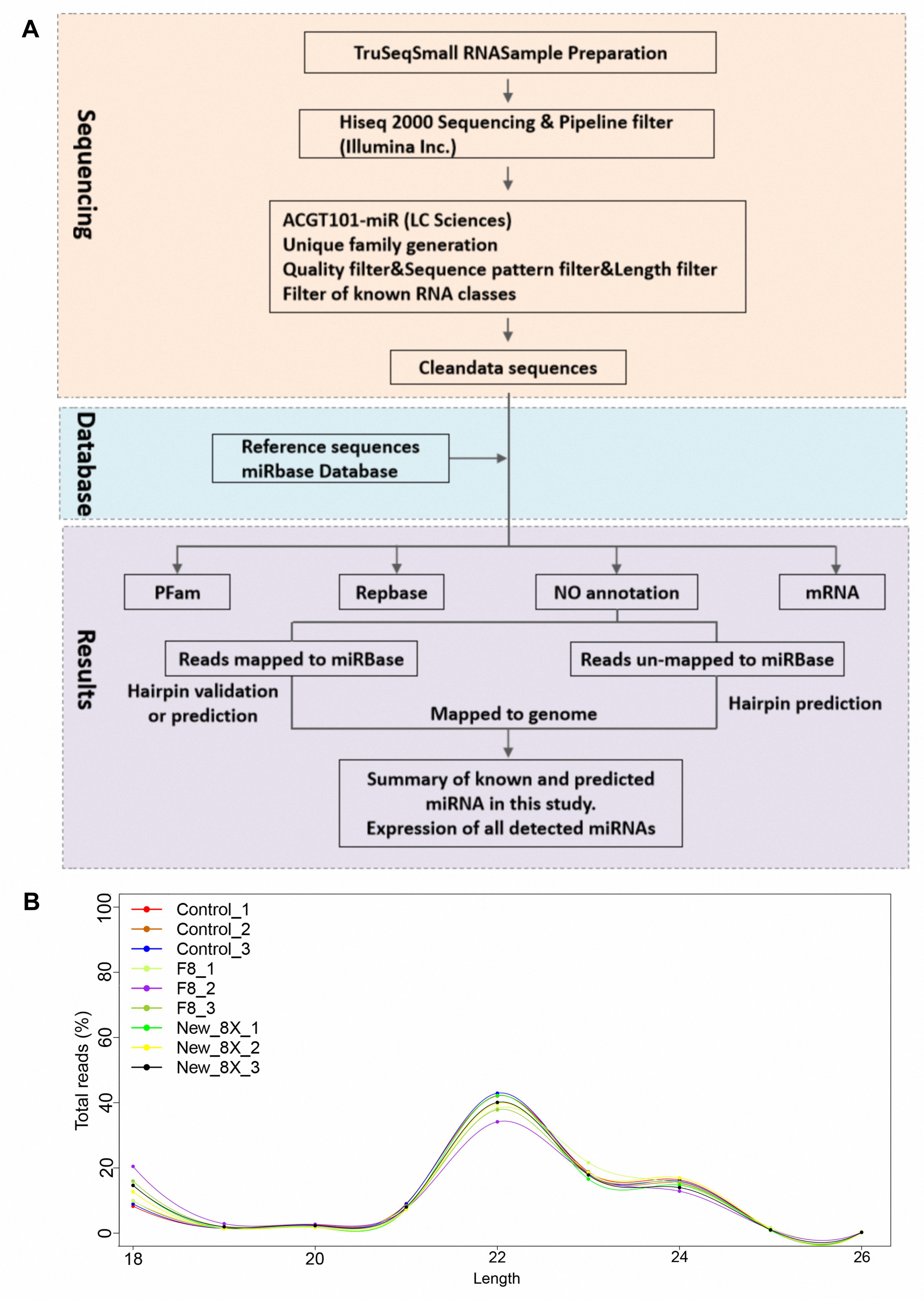

Supplement: Supplementary Figure S1 — Workflow of miRNA sequencing. [file Image_1.tif]

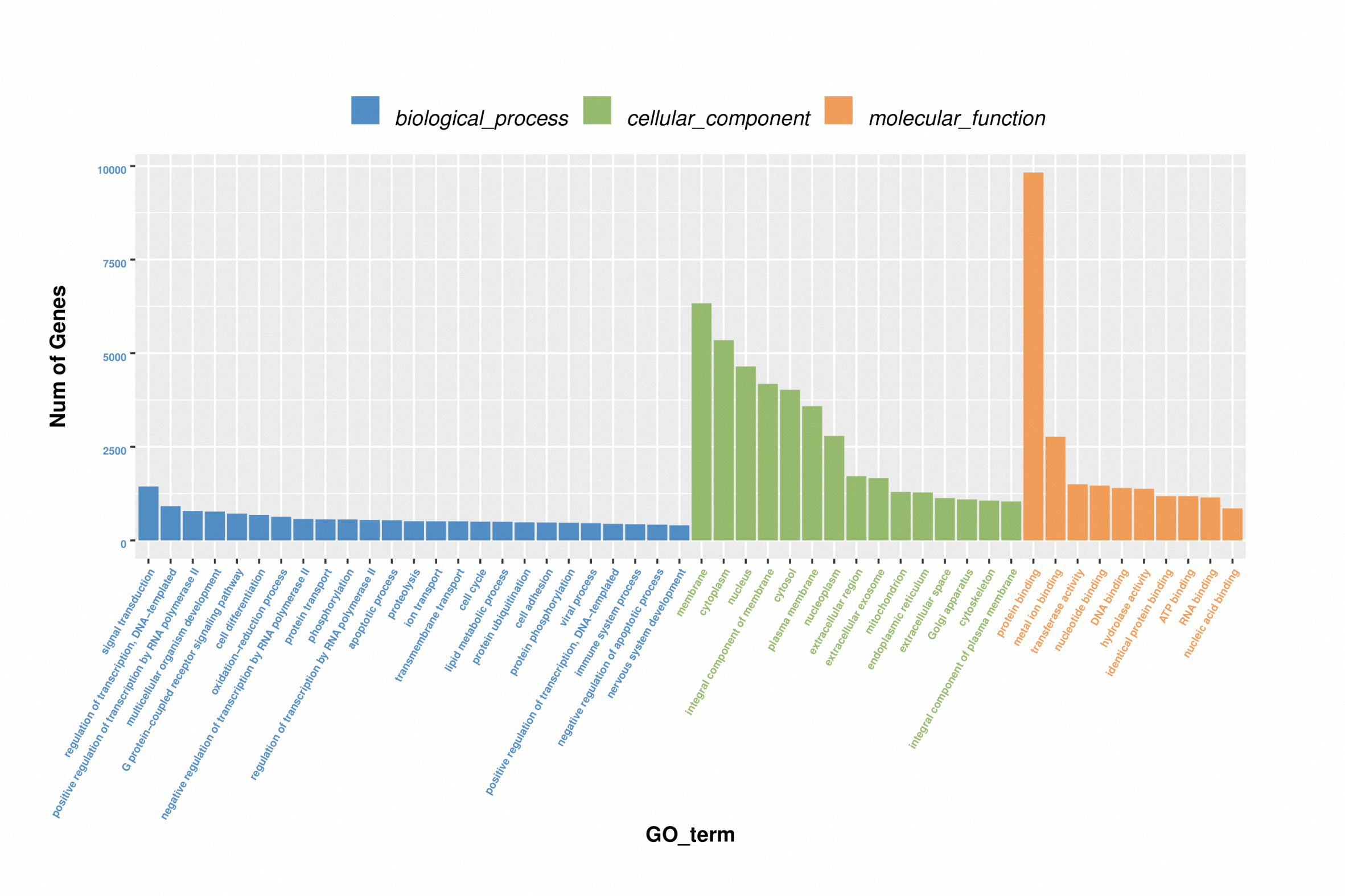

Supplement: Supplementary Figure S2 — GO analysis of miRNAs. [file Image_2.tif]

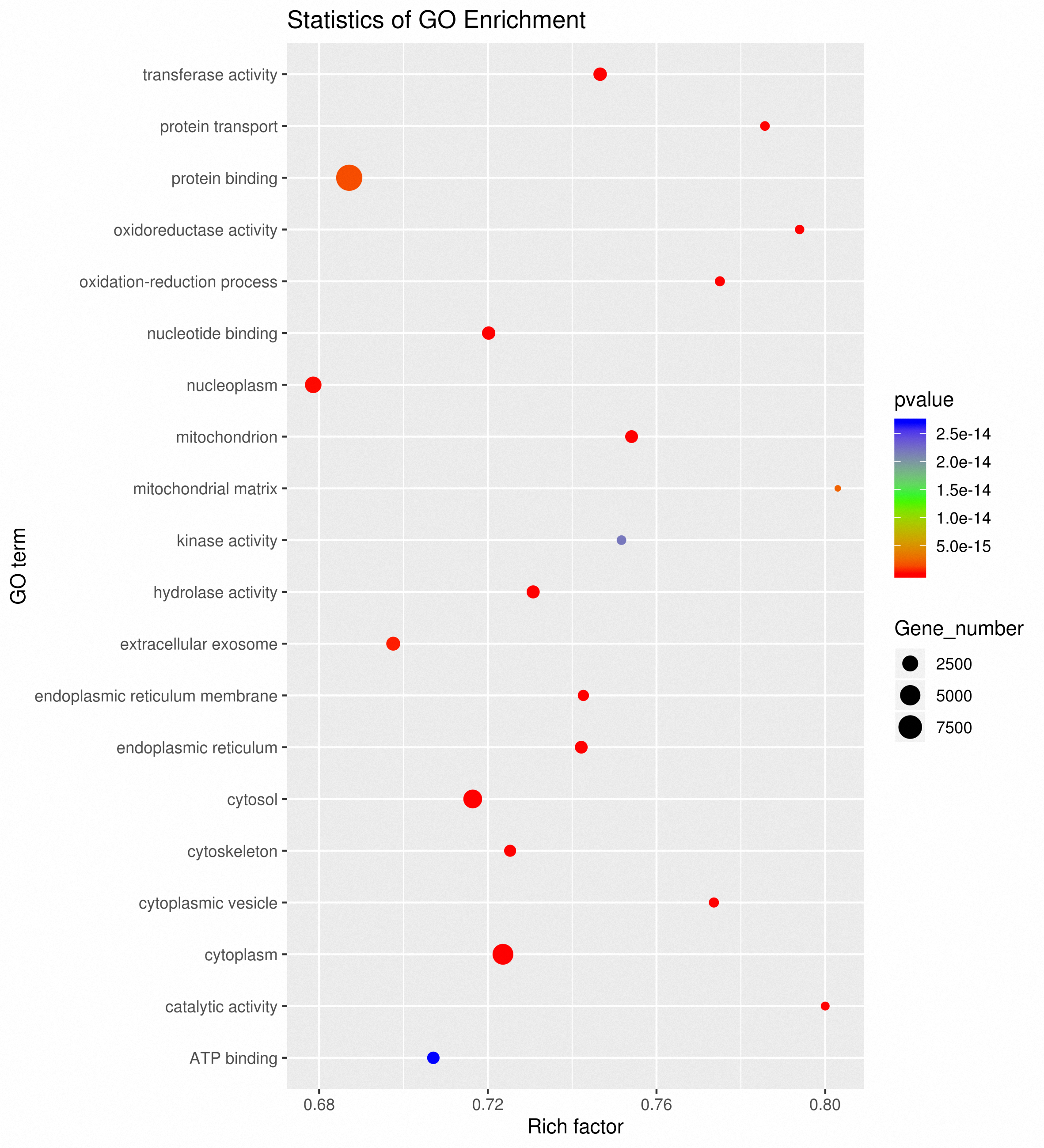

Supplement: Supplementary Figure S3 — GO enrichment of different miRNAs between F8 and 8X. [file Image_3.tif]

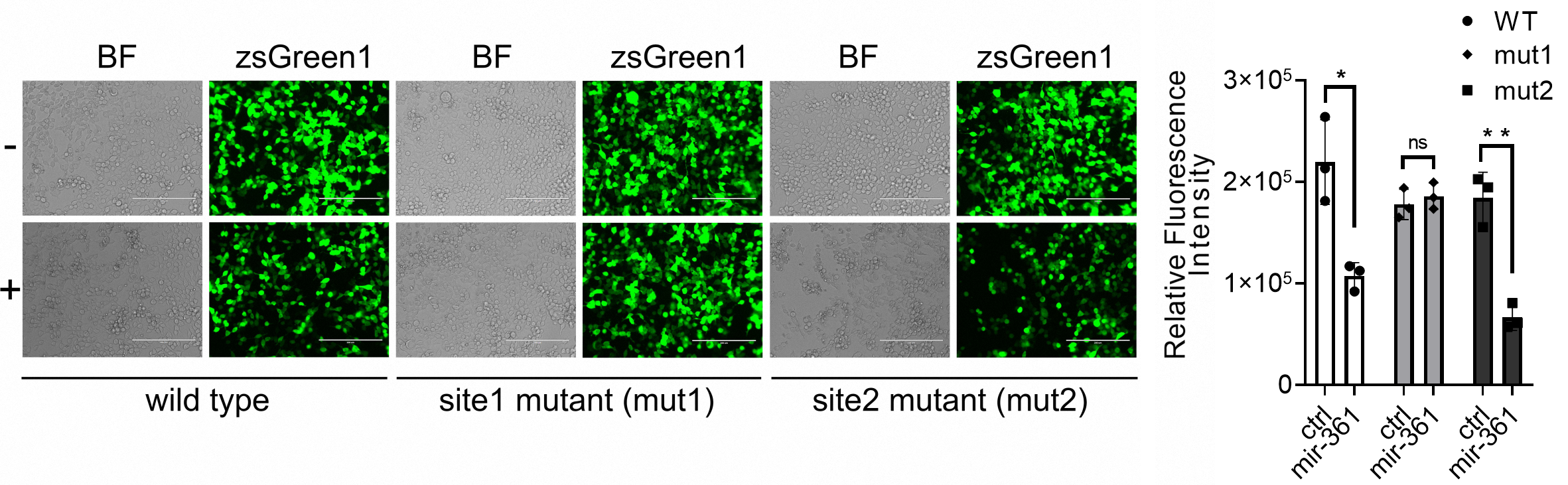

Supplement: Supplementary Figure S5 — Identification for target site of miR-361-3p on 3′UTR of TSPAN1 in HEK293T. [file Image_5.tif]
